# Association Between Statin Use and Prognosis of Breast Cancer: A Meta-Analysis of Cohort Studies

**DOI:** 10.3389/fonc.2020.556243

**Published:** 2020-10-16

**Authors:** Hui Lv, Ding Shi, Min Fei, Yu Chen, Fei Xie, Zhuoyan Wang, Ying Wang, Peiying Hu

**Affiliations:** ^1^Health Promotion Center, Zhejiang Provincial People's Hospital, People's Hospital of Hangzhou Medical College, Hangzhou, China; ^2^State Key Laboratory for Diagnosis and Treatment of Infectious Diseases, National Clinical Research Center for Infectious Diseases, Collaborative Innovation Center for Diagnosis and Treatment of Infectious Diseases, The First Affiliated Hospital, College of Medicine, Zhejiang University, Hangzhou, China

**Keywords:** breast cancer, statin, recurrence, mortality, meta-analysis

## Abstract

**Background:** Statin, a lipid-lowering drug, has been suggested to confer anticancer efficacy. However, previous studies evaluating the association between statin use and prognosis in breast cancer showed inconsistent results. A meta-analysis was performed to evaluate the association between statin use and clinical outcome in women with breast cancer.

**Methods:** Cohort studies comparing recurrence or disease-specific mortality in women with breast cancer with and without using of statins were identified by search of PubMed, Embase, and Cochrane's Library databases. A random-effect model, incorporating the inter-study heterogeneity, was used to combine the results. Subgroup analyses were performed to evaluate the influences of study characteristics on the outcomes

**Results:** Seventeen cohort studies with 168,700 women with breast cancer were included. Pooled results showed that statin use was significantly associated with a lower risk of breast cancer recurrence (adjusted hazard ratio [HR] = 0.72, *p* < 0.001) and breast cancer mortality (HR = 0.80, *p* < 0.001). Subgroup analysis showed that timing of statin use, statin type, study design, sample size, or quality score did not significantly affect the outcomes. However, statin use was associated with more remarkably reduced breast cancer recurrence in studies with mean follow-up duration ≤ 5 years (HR = 0.55, *p* < 0.001) than that in studies of >5 years (HR = 0.83, *p* = 0.01).

**Conclusions:** Statin use is associated with reduced recurrence and disease-specific mortality in women with breast cancer. These results should be validated in randomized controlled trials.

## Introduction

Although advances have been achieved in the prevention and treatment of breast cancer in recent decades, the disease remains one of the most common malignancies in women (1, 2). It has been reported that ~1.4 million women are diagnosed as breast cancer each year all over the world, and breast cancer remains an important cause of mortality in women (3, 4). The 3-hydroxy-3-methylglutaryl CoA (HMG-CoA) reductase inhibitors, also known as statins, are the most commonly use lipid-lowering medications which have become a cornerstone for the prevention and treatment of atherosclerotic cardiovascular diseases (5). Accumulating evidence revealed that statins have various potential pharmacological effects besides their lipid-lowering efficacy, such as anti-inflammation, anti-proliferation, and anti-invasion, pro-apoptosis, immunomodulation, which are all involved in the pathogenesis of cancer (6, 7). These findings highlight the potential role of statins as anticancer agents (8). Although previous studies generally did not show that statin use is related with reduced risk of breast cancer incidence (9–11), some cohort studies showed that compared with the non-users, users of statin with breast cancer may have better clinical outcomes (12–17). However, other cohort studies did not show that statin use in women with breast cancer was associated with improved prognosis (18–28). Although several meta-analyses have been performed to evaluate the association between statin use and prognosis in women with breast cancer (29–32), only studies published before 2017 were included, and the limited number of studies prevented a comprehensive evaluation of the impacts of study characteristics on the outcomes. Therefore, we aimed to perform an updated meta-analysis regarding the association between statin use and prognosis in breast cancer, by incorporating of the recently published cohorts that were not included in previous meta-analyses (17, 24–28). The relative large number of available studies enables us to perform comprehensive analyses regarding the influences of study characteristics on the outcomes.

## Methods

The meta-analysis was designed and performed in accordance with the MOOSE (Meta-analysis of Observational Studies in Epidemiology) (33) and Cochrane's Handbook (34) guidelines.

### Literature Search

Electronic databases of PubMed, Embase, and the Cochrane's Library were systematically searched using the combination of the following terms: (1) “statin” OR “3-hydroxy-3-methyl-glutaryl CoA reductase inhibitor” OR “CS-514” OR “simvastatin” OR “atorvastatin” OR “fluvastatin” OR “lovastatin” OR “rosuvastatin” OR “pravastatin” OR “pitavastatin”; (2) “breast cancer”; and (3) “survival” OR “prognosis” OR “mortality” OR “death” OR “recurrence” OR “surgery” OR “operation.” The search was limited to human studies with no restriction of publication language. The reference lists of original and review articles were also manually analyzed. The final literature search was performed on February 24, 2020.

### Study Selection

Studies were included if they met the following criteria: (1) published as full-length articles; (2) designed as cohort studies with the minimal follow-up duration of 1 year; (3) included women with breast cancer; (4) use of statin was identified as exposure of interest; (5) documented the incidence of breast cancer recurrence or breast cancer mortality during follow-up; and (6) reported the adjusted hazard ratios (HRs, at least adjusted for age) and their corresponding 95% confidence intervals (CIs) for the above outcomes in women with breast cancer with and without the use of statin. Reviews, editorials, preclinical studies, and non-cohort studies were excluded.

### Data Extracting and Quality Evaluation

Literature search, data extraction, and study quality assessment were independently performed by two authors according to the predefined inclusion criteria. If inconsistencies occurred, discussion with the corresponding author was suggested to resolve these issues. The following data were extracted: (1) name of the first author, publication year, country, and study design; (2) characteristics, number, and mean age of women with breast cancer, definition and timing of statin use, and follow-up period; and (3) number of cases with breast cancer recurrence and breast cancer mortality, and the adjusted variables when presenting the HRs. The quality of each study was evaluated using the Newcastle-Ottawa Scale (NOS) (35). This scale ranges from 1 to 9 stars and judges the quality of each study regarding three aspects: selection of the study groups; the comparability of the groups; and the ascertainment of the outcome of interest.

### Statistical Analyses

The associations between statin use and breast cancer recurrence and mortality were measured by HRs in this study. To stabilize its variance and normalized the distribution, HR data and its corresponding stand error (SE) from each study were logarithmically transformed (34). The Cochrane's Q-test was performed to evaluate the heterogeneity among the include cohort studies (34, 36), and an *I*^2^ statistic was also calculated. A significant heterogeneity was considered if *I*^2^ > 50%. A random-effect model was used to pool the results since this model has been indicated to incorporate of the potential heterogeneity among the included studies and therefore could provide a more generalized result. Sensitivity analysis by omitting one study at a time was performed to evaluate the stability of the results (34). Predefined subgroup analysis was used to evaluate the potential influences of study characteristics on the outcome (37), including study design, sample size, follow-up duration, timing of statin use, category of statins, exposure time to statins, adjustment of menopausal status, hormonal receptor status, or comorbidities, and quality score of the study. Medians of the continuous variables were used as cut-off values for defining of subgroups. Because different cut-off values were applied in studies when analyzing the statin exposure time on the outcomes (16, 18, 20, 21, 25, 27), we compared the HRs in subgroups with the shortest and the longest exposure time. Potential publication bias was assessed by visual inspection of the symmetry of the funnel plots and the Egger regression test (38). The RevMan (Version 5.1; Cochrane Collaboration, Oxford, UK) and STATA software were used for the statistics.

## Results

### Literature Search

**Figure 1:**
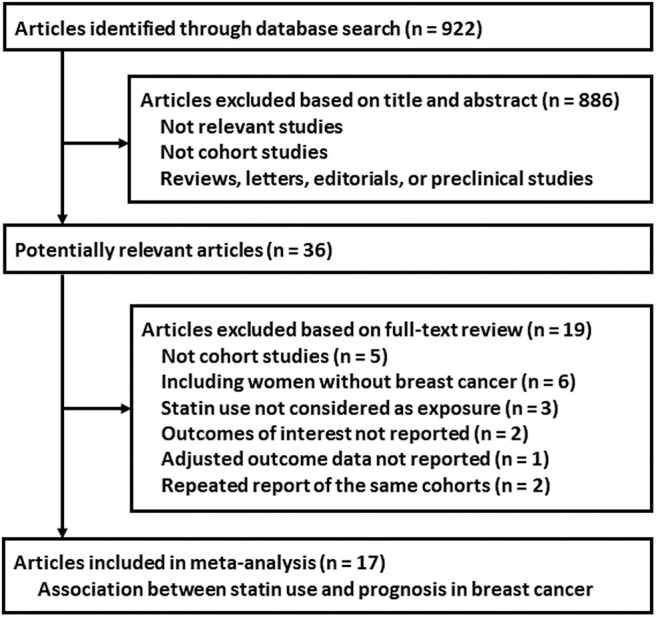
Flowchart of database search and study identification.

The flowchart of database search was shown in Figure 1. Briefly, 922 studies were obtained from database search, and 886 of them were excluded primarily because they were not relevant to the aim of the meta-analysis. For the remaining 36 studies that underwent full text review, 19 were further excluded for the reasons listed in Figure 1. Finally, 17 cohort studies were included (12–28).

### Study Characteristics and Quality

Overall, this meta-analysis included 17 cohort studies (12–28) with 168,700 women with breast cancer. The characteristics of the included cohorts were shown in Table 1. Eight of them were prospective cohort studies (12, 14, 16–18, 25, 26, 28), while the other nine were retrospective (13, 15, 19–23, 25, 27). Women with breast cancer of different clinical stages were included. Statin use was defined as statin exposure before, at, and after the diagnosis of breast cancer in different studies. The follow-up durations varied from 3 to 7 years. Potential confounding factors, including age, menopausal status, cancer stage at diagnosis, histological grade, hormonal receptor status, comorbidities, and concurrent anticancer treatments were adjusted to varying degrees in the included studies. The qualities of the included follow-up studies were generally good, with the NOS ranging from 6 to 9 (Table 2).

**Table 1:** Characteristics of the included cohort studies.

**Study**	**Country**	**Design**	**Patient characteristics**	**Sample size**	**Mean age**	**Timing of statin use**	**Follow-up duration**	**Outcomes reported (*n*)**	**Outcome validation**	**Variables adjusted**
					**Years**		**Years**			
Kwan et al. (18)	the US	PC	Stage I-IIIa BC women after completed treatment	1,811	58.4	Any statin use of >100 cDDD after BC diagnosis	5.0	Recurrence (210)	Medical record	Age at diagnosis, race, BMI, stage of BC, and TMX treatment
Chae et al. (13)	the US	RC	Stage II-III BC women after curative treatment	703	59.1	Any statin use of >180 cDDD after BC diagnosis	4.6	Recurrence (149)	Medical record	Age, race, menopausal status, family history, smoking history, DM, HR status, and hormonal therapy
Ahern et al. (12)	Denmark	PC	Stage I-III BC women after surgery	18,769	NR	Any statin use after BC diagnosis	6.8	Recurrence (3,419)	Medical record	Age, menopausal status, histological grade, ER status, hormonal therapy, cancer treatment, and concurrent use of other medications
Nielsen et al. (14)	Denmark	PC	BC women after treatment	45,652	NR	Any statin use within 2 years before the diagnosis of BC	3.6	BC-mortality (11,960)	Medical record	Age, education, study area, stage of BC, cancer treatments, and comorbidities
Botteri et al. (19)	Italy	RC	Postmenopausal stage I-III TNBC women after treatments	800	59.8	Any statin use at the diagnosis of BC	5.7	Recurrence (212) and BC-mortality (147)	Medical record	Age, BMI, stage of BC, cancer treatments, comorbidities, and concurrent medications
Brewer et al. (15)	the US	RC	Women with stage III IBC	723	49.6	Any statin use at the diagnosis of BC	2.9	Recurrence (433) and BC-mortality (366)	Medical record	Age, BMI, stage of IBC, HR status, comorbidities, cancer treatment and concurrent medications
Boudreau et al. (20)	the US	RC	Women with stage I-II BC	4,216	63.0	Any statin use after the diagnosis of BC	6.3	Recurrence (415)	Medical record	Age, BMI, BC stage, HR status, menopausal status, CCI, DM, cancer treatments and concurrent medications
Murtola et al. (16)	Finland	PC	Women with stage I-IV BC	31,236	58.6	Any statin use before, at, or after the diagnosis of BC	3.3	BC-mortality (3,619)	Medical record	Age, tumor stage, morphology and treatment selection
Cardwell et al. (21)	UK	RC	Women with stage I-IV BC 1 year after diagnosis	17,880	NR	Any statin use within 1 year before or during follow-up after the diagnosis of BC	5.7	BC-mortality (2,222)	Medical record	Age, cancer treatment, hormonal therapy, comorbidities, and concurrent medications
Sakellakis et al. (23)	Greece	RC	Women with stage I-III BC after treatment	610	56.8	Any statin use at the diagnosis of BC	3.4	Recurrence (133)	Medical record	Age, tumor stage, and HR status
Mc Menamin et al. (22)	Scotland	RC	Women with stage I-IV BC after treatment	15,140	NR	Statin use within 1 year before or during follow-up after diagnosis of BC	4.	BC-mortality (1,190)	Medical record	Age, cancer stage and grade, cancer treatments, comorbidities, socioeconomic status and use of aspirin
Smith et al. (25)	Ireland	PC	Women with stage I-III BC after treatment	6,314	68.1	Any statin use before or after the diagnosis of BC	4.9	BC-mortality (773)	Medical record	Age, smoking status, comorbidity score, tumor stage and grade, HR status, cancer treatments, hormonal therapy, and concurrent medications
Shaitelman et al. (24)	the US	RC	Women with stage I-III TNBC after treatments	869	51.0	Any statin use after the diagnosis of BC	6.3	Recurrence (151)	Medical record	Age, BMI, tumor stage and grade, and cancer treatments
Tryggvadottir et al. (26)	Sweden	PC	Women with stage I-III BC	985	61.0	Any statin use after the diagnosis of BC	7	Recurrence (150)	Medical record	Ag, BMI, tumor stage and histological grade, ER status, alcoholism, and treatments
Li et al. (27)	the US	RC	Women with stage I-III BC	1,523	64.9	Any statin use after the diagnosis of BC	6.9	Recurrence (219)	Medical record	Ag, BMI, tumor stage, HR status, and CCI
Borgquist et al. (17)	Sweden	PC	Women > 40 years with BC	20,559	69.0	Any statin use before or during follow-up after the diagnosis of BC	5.1	BC-mortality (2,669)	Medical record	Age, tumor stage, DM, and treatments
Bjarnadottir et al. (28)	Sweden	PC	Women with stage I-III BC	910	65.5	Any statin use before or during follow-up after the diagnosis of BC	5.4	BC-mortality (37)	Medical record	Age, tumor stage and histological grade, ER status, and cancer treatments

*BC, breast cancer; NOS, the Newcastle-Ottawa Scale; US, United States; UK, United Kingdom; TNBC, triple-negative breast cancer; IBC, inflammatory breast cancer; NR, not reported; PC, prospective cohort; RC, retrospective cohort; cDDD, cumulative defined daily dose; BMI, body mass index; DM, diabetes mellitus; HER-2, human epidermal growth factor receptor-2; HR, hormone receptor; ER, estrogen receptor; PR, progesterone receptor; TMX, tamoxifen; CCI, Charlson comorbidity index*.

**Table 2:** Details of study quality evaluation via the Newcastle-Ottawa Scale.

**Study**	**Representativeness of the exposed cohort**	**Selection of the non-exposed cohort**	**Ascertainment of exposure**	**Outcome not present at baseline**	**Control for age**	**Control for other confounding factors**	**Assessment of outcome**	**Enough long follow-up duration**	**Adequacy of follow-up of cohorts**	**Total**
Kwan et al. (18)	0	1	1	1	1	1	1	1	0	7
Chae et al. (13)	0	1	1	1	1	1	1	1	0	7
Ahern et al. (12)	1	1	1	1	1	1	1	1	1	9
Nielsen et al. (14)	1	1	1	1	1	1	1	0	1	8
Botteri et al. (19)	0	1	1	1	1	1	1	1	0	7
Brewer et al. (15)	1	1	1	1	1	1	1	0	0	7
Boudreau et al. (20)	0	1	1	1	1	1	1	1	0	7
Murtola et al. (16)	1	1	1	1	1	1	1	1	1	9
Cardwell et al. (21)	1	1	1	1	1	1	1	1	0	8
Sakellakis et al. (23)	0	1	0	1	1	1	1	1	0	6
Mc Menamin et al. (22)	0	1	1	1	1	1	1	1	0	7
Smith et al. (25)	0	1	1	1	1	1	1	1	1	8
Shaitelman et al. (24)	0	1	1	1	1	1	1	1	0	7
Tryggvadottir et al. (26)	0	1	1	1	1	1	1	1	0	7
Li et al. (27)	0	1	1	1	1	1	1	1	1	7
Borgquist et al. (17)	1	1	1	1	1	1	1	1	1	9
Bjarnadottir et al. (28)	0	1	1	1	1	1	1	1	1	8

### Association Between Statin Use and Breast Cancer Recurrence

**Figure 2:**
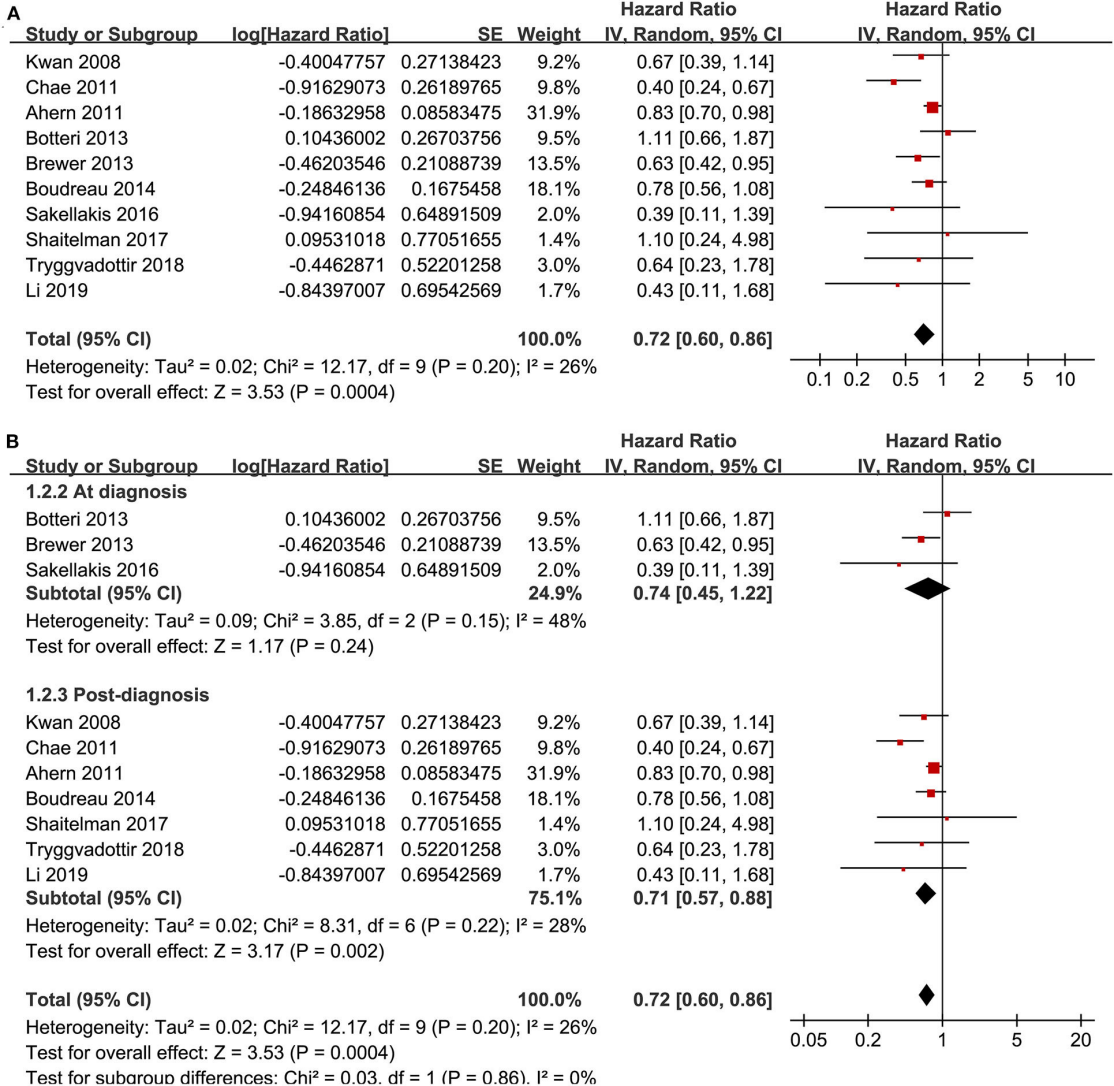
Meta-analysis for the association between statin use and recurrence of breast cancer; **(A)** main meta-analysis; and **(B)** stratified analysis by the timing of statin use.

**Figure 3:**
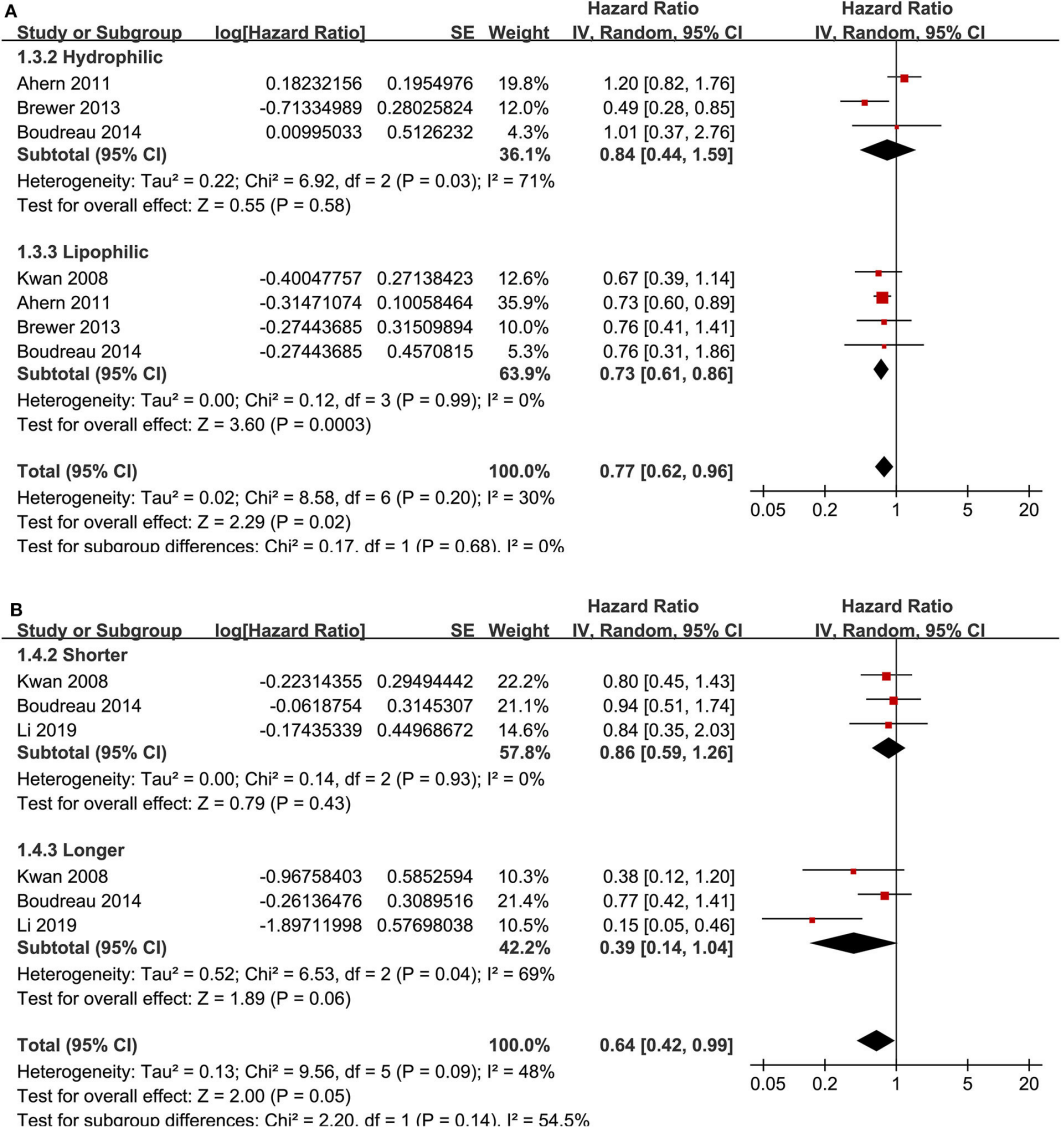
Stratified analyses for the association between statin use and recurrence of breast cancer. **(A)** stratified analysis by the category of statin; and **(B)** stratified analysis by the exposure time of statin.

Ten cohort studies (12, 13, 15, 18–20, 23, 24, 26, 27) reported the association between statin use and recurrence of breast cancer. In the original manuscript from Li, the HR results for breast cancer recurrence were reported according to the time of statin use (<3, 3–5, and >5 years) (27). These results were firstly pooled with a random-effect model to generate a data of HR for women with statin use of any time compared to non-users, and then the pooled HR was included in the meta-analysis. The heterogeneity among these studies was not significant (P for Cochrane's Q-test = 0.20, *I*^2^ = 26%). Pooled results with a random-effect model showed that statin use was associated with a significantly reduced breast cancer recurrence (adjusted HR = 0.72, 95% CI: 0.60 to 0.86, *p* < 0.001; Figure 2A). Sensitivity analysis by omitting one study at a time showed similar results (HR: 0.68–0.79, *p* all <0.05). Stratified analyses showed that the results were not statistically different between studies with statin use at the diagnosis or after the diagnosis of breast cancer (HR: 0.74 vs. 0.71, *p* for subgroup difference = 0.86; Figure 2B), between hydrophilic or lipophilic statin (HR: 0.84 vs. 0.73, *p* for subgroup difference = 0.68; Figure 3A), or between women with shorter or longer statin exposure (HR: 0.86 vs. 0.39, *p* for subgroup difference = 0.14; Figure 3B). In addition, subgroup analysis also showed that difference in study design, sample size, NOS, and adjustment of menopausal status, hormonal receptor status, or comorbidities did not significantly affect the results (*p* for subgroup difference all >0.10; Table 3). However, statin use was associated with a more remarkably reduced breast cancer recurrence in studies with mean follow-up duration ≤ 5 years (HR = 0.55, *p* < 0.001) than that in studies with mean follow-up duration >5 years (HR = 0.83, *p* = 0.01; *p* for subgroup difference = 0.009; Table 3).

**Table 3:** Subgroup analyses.

	**BC recurrence**					**BC mortality**				
**Study characteristics**	**Datasets number**	**HR (95% CI)**	***I*^**2**^ (%)**	**P for subgroup effect**	**P for subgroup difference**	**Datasets number**	**HR (95% CI)**	***I*^**2**^ (%)**	**P for subgroup effect**	**P for subgroup difference**
**Study design**										
PC	3	0.81 [0.69, 0.95]	0	0.009		5	0.76 [0.63, 0.91]	76	0.004	
RC	7	0.67 [0.50, 0.90]	39	0.008	0.26	4	0.87 [0.78, 0.97]	0	0.009	0.23
**Sample size**										
<1,000	6	0.64 [0.44, 0.94]	42	0.02		3	0.91 [0.63, 1.31]	0	0.60	
≥1,000	4	0.80 [0.69, 0.93]	0	0.003	0.29	6	0.79 [0.70, 0.90]	71	<0.001	0.51
**Follow-up duration (years)**										
≤ 5	4	0.55 [0.42, 0.72]	0	<0.001		5	0.79 [0.66, 0.95]	77	0.01	
>5	6	0.83 [0.72, 0.96]	0	0.01	0.009	4	0.81 [0.73, 0.91]	0	<0.001	0.80
**Adjustment of menopausal status**										
Yes	4	0.75 [0.56, 1.01]	66	0.06		3	0.71 [0.44, 1.14]	68	0.15	
No	6	0.63 [0.47, 0.84]	0	0.002	0.40	6	0.85 [0.80, 0.91]	0	<0.001	0.44
**Adjustment of hormonal receptor status**										
Yes	9	0.72 [0.58, 0.88]	33	0.002		4	0.86 [0.71, 1.04]	0	0.12	
No	1	0.67 [0.39, 1.14]	—	0.14	0.81	5	0.78 [0.68, 0.91]	77	0.001	0.47
**Adjustment of comorbidities**										
Yes	3	0.76 [0.47, 1.22]	42	0.25		5	0.87 [0.81, 0.94]	0	<0.001	
No	7	0.70 [0.56, 0.87]	31	0.002	0.76	4	0.71 [0.55, 0.92]	74	0.009	0.13
**NOS Score**										
6–7	8	0.68 [0.51, 0.89]	28	0.006		2	0.88 [0.77, 1.01]	0	0.07	
8–9	2	0.77 [0.61, 0.98]	32	0.03	0.48	7	0.78 [0.68, 0.90]	64	<0.001	0.25

*BC, breast cancer; HR^a^, hazard ratio; CI, confidence interval; PC, prospective cohort; RC, retrospective cohort; NOS, the Newcastle-Ottawa Scale*.

### Association Between Statin Use and Breast Cancer Mortality

**Figure 4:**
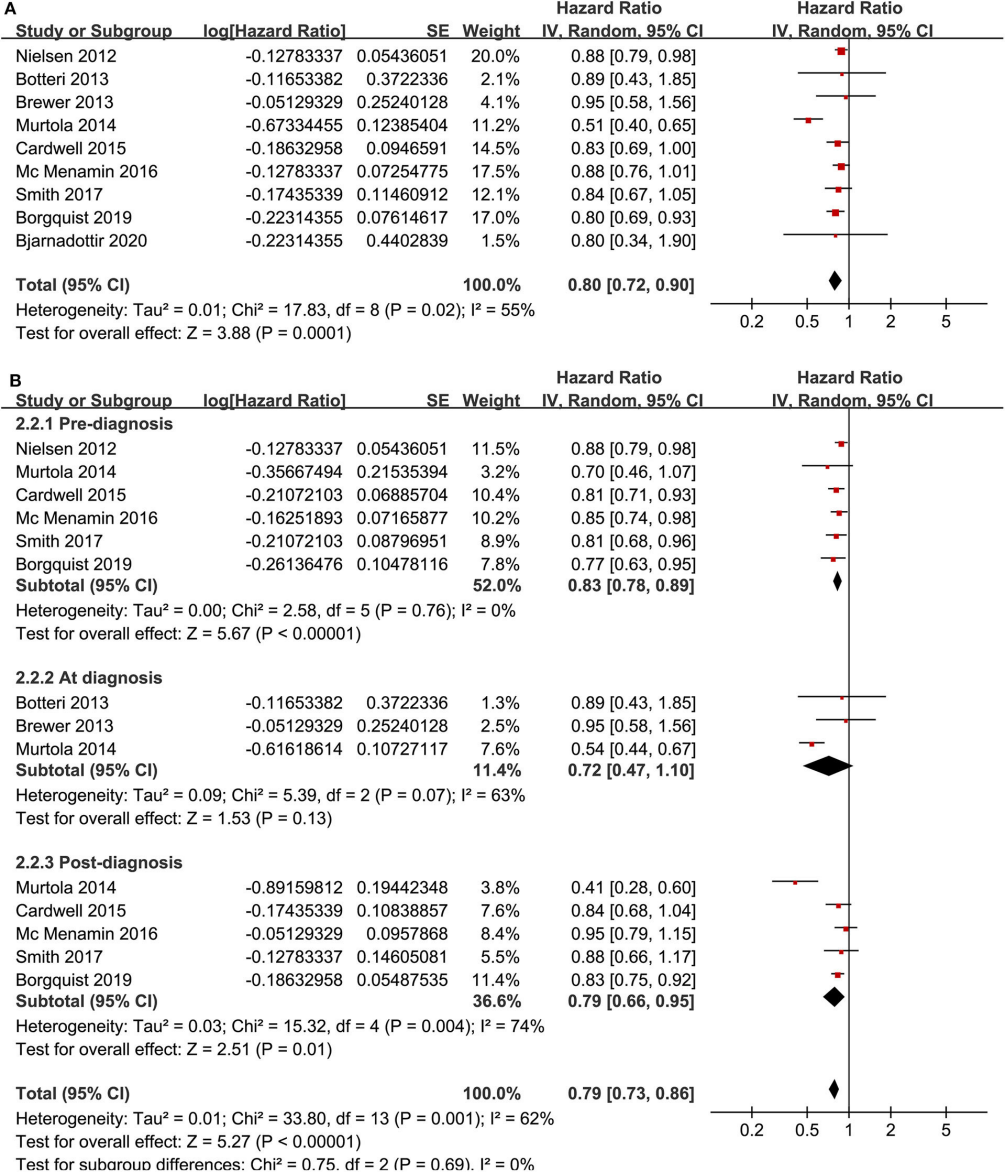
Meta-analysis for the association between statin use and disease-specific mortality of breast cancer; **(A)** main meta-analysis; and **(B)** stratified analysis by the timing of statin use.

**Figure 5:**
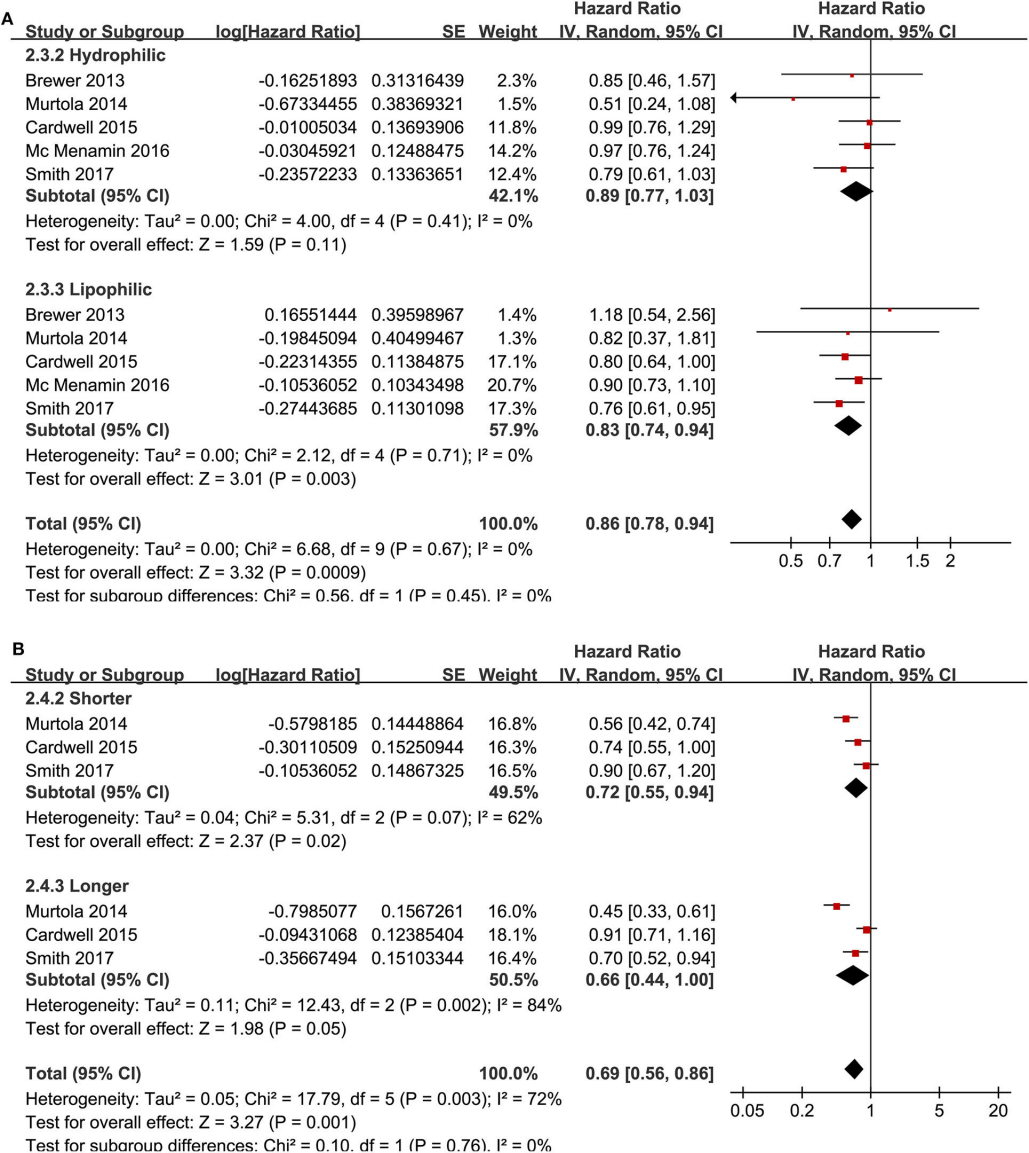
Stratified analyses for the association between statin use and disease-specific mortality of breast cancer. **(A)** stratified analysis by the category of statin; and **(B)** stratified analysis by the exposure time of statin.

Meta-analysis of nine cohort studies (14–17, 19, 21, 22, 25, 28) showed that statin use was associated with a significantly reduced risk of breast cancer mortality (adjusted HR = 0.80, 95% CI: 0.72 to 0.90; *p* < 0.001) with significant heterogeneity (*I*^2^ = 55%; Figure 4A). Sensitivity analysis by omitting one study at a time showed similar results (HR: 0.79–0.86, *p* all <0.05). Stratified analyses showed that the results were not statistically different for studies with statin use before, at, or after the diagnosis of breast cancer (HR: 0.74, 0.72, and 0.79, p for subgroup difference = 0.69; Figure 4B), between hydrophilic or lipophilic statin (HR: 0.89 vs. 0.83, p for subgroup difference = 0.45; Figure 5A), or between women with shorter or longer statin exposure (HR: 0.72 vs. 0.66, *p* for subgroup difference = 0.76; Figure 5B). Furthermore, subgroup analysis also showed that differences in study design, sample size, follow-up duration, NOS, adjustment of menopausal status, hormonal receptor status, or comorbidities did not significantly affect the results (*p* for subgroup difference all >0.10; Table 3).

### Publication Bias

**Figure 6:**
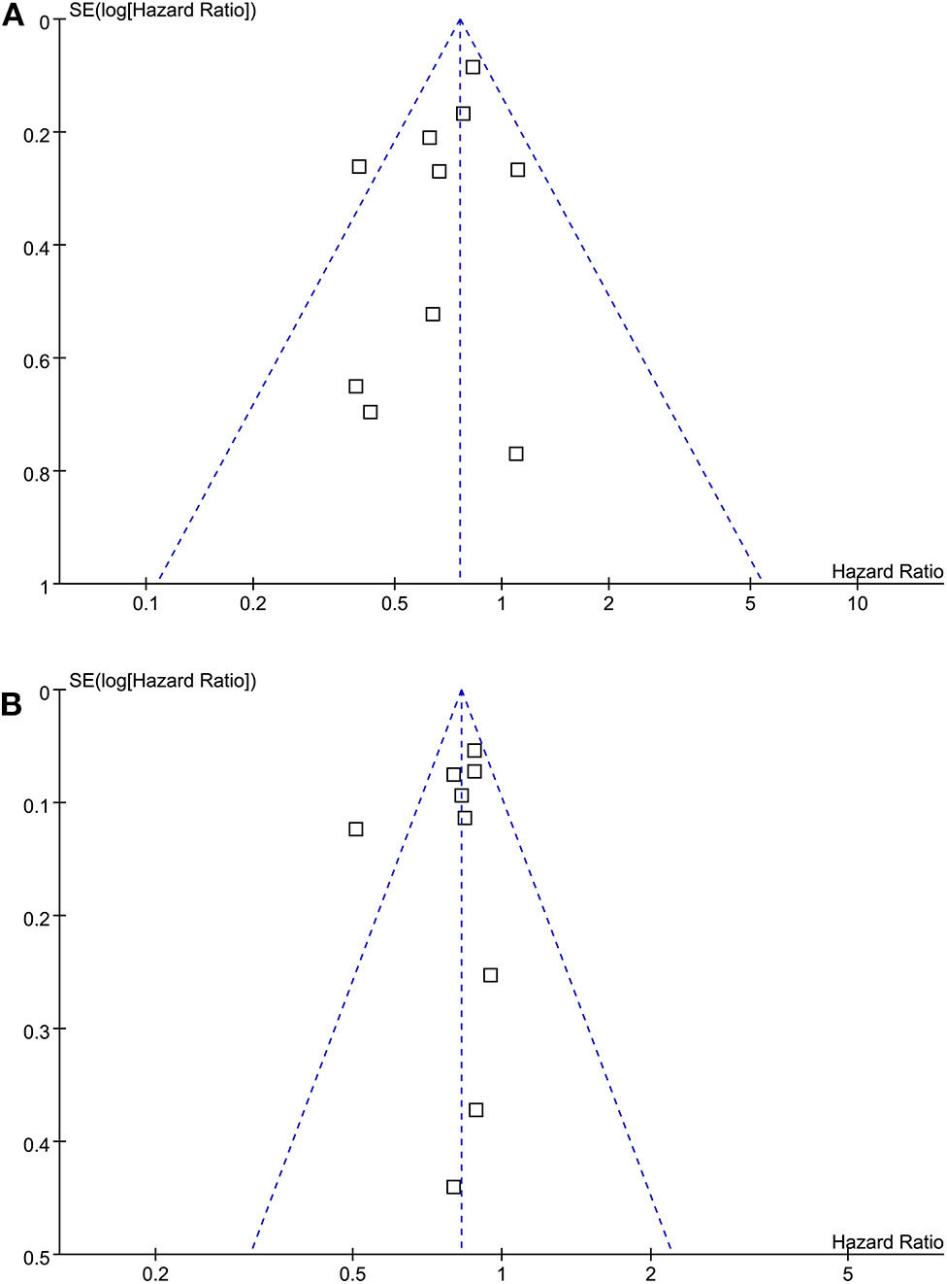
Funnel plots for the meta-analyses of the association between statin use and prognosis of breast cancer. **(A)** recurrence of breast cancer; and **(B)** breast cancer mortality.

The funnel plots for the associations between statin use and breast cancer recurrence and mortality were shown in Figures 6A,B. The plots were symmetrical on visual inspection, suggesting low risks of publication biases. Results of Egger's regression tests also showed similar results (*p* = 0.328 and 0.384, respectively).

## Discussion

In this meta-analysis of cohort studies, we found that compared to the non-users, statin use was associated with significant lower recurrence and disease-specific mortality in women with breast cancer, even after adjustment of potential confounding factors including age, cancer stage, and anticancer treatments. Subgroup analysis showed that study characteristics such as timing of statin use, statin type, statin exposure time, study design, sample size, quality score, adjustment of menopausal status, hormonal receptor status, or comorbidities did not seem to significantly affect the association between statin use and improved prognosis in women with breast cancer. However, a more remarkably reduced breast cancer recurrence was observed in studies with shorter follow-up duration (≤5 years) compared to that in studies with longer follow-up duration (>5 years). Taken together, these findings suggest that statin use is associated with reduced recurrence and disease-specific mortality in women with breast cancer, which supports the implementation of a randomized clinical trial.

Several meta-analyses have been performed to evaluate the association between statin use and prognosis in women with breast cancer (29–32). Although results of these meta-analyses were generally consistent the overall results of our meta-analysis, these studies only included five to eight cohort studies, which prevented subsequent analyses for the influences of study characteristics on the outcomes. A previous meta-analysis by Liu et al. published in 2017 showed that the relationship between statins use and breast cancer was remarkable in studies with lipophilic statins and statin exposure of <4 years (32). However, only seven cohorts were included in this meta-analysis, and the authors used the mean follow-up year as a reflection of statin exposure year, which made the results less reliable (32). Compared to previous meta-analyses, our study has the following strengths. Firstly, we included up-to-date evidence from related cohort studies, which included 17 studies with 168,700 women with breast cancer. This large number of studies enables us to perform comprehensive subgroup analyses based on the data of study level. Secondly, only studies with multivariate analyses were included. Therefore, our study results indicated that statin use was independently associated with improved prognosis in women with breast cancer. Thirdly, sensitivity analyses were used to evaluate the stability of the results, which showed that the overall meta-analysis results were not affected by either of the included study. Finally, results of subgroup analyses suggested that statin was associated with a more remarkably reduced breast cancer recurrence in studies with shorter follow-up duration (≤5 years) compared to that in studies with longer follow-up duration (>5 years). One possible explanation for this finding may be that compared to short-term recurrence, mechanisms responsible for the long-term recurrence of breast cancer could be more complicated, and the potential protective efficacy of statins might be weakened. Moreover, it has been reported that triple negative breast cancer tends to recur in <5 years whereas hormone receptor positives have longer periods of dormancy (39). The difference in molecular subtype of breast cancer may be accounted for the subgroup results. However, we could not confirm this hypothesis because the molecular subtype of breast cancer was generally not reported in studies included in the subgroup analysis according to statin exposure time. Besides, it has to be mentioned that since the exposure time of statin in each study is not necessarily correlated with the follow-up time. Therefore, the finding of the subgroup analysis may be less clinically relevant.

The mechanisms underlying the potential association between statin use and lower breast cancer recurrence and mortality remain largely unknown at current stage. A previous cohort study including 191 Korean women with breast cancer who underwent resection showed that a higher tumor expression of HMG-CoA reductase was associated with poor disease-free survival, which suggests that the potential benefit of statin on clinical outcomes in breast cancer may involve its pharmacological effect on HMG-CoA reductase inhibiting (40). A recent study in Swedish women with breast cancer who were on statins also showed similar finding (28). More direct evidence comes from a recent experimental study, which showed that induction of tumor expression of HMG-CoA reductase led to resistance to statin induced deaths of breast cancer cells (41), which further demonstrated that the benefits of statins in breast cancer are at least partially depending on their inhibition of HMG-CoA reductase. Besides, preclinical studies also suggest that statin may exert anticancer efficacy in breast cancer via other mechanisms. It has been suggested that inhibition of protein prenylation involved in signaling pathways of carcinogenesis and cancer progression may be halted as a downstream effect of HMGCR inhibition by statins (42). In addition, simvastatin was shown to inhibit breast tumor angiogenesis via impeding hypoxia-inducible factor-1α-induced pro-angiogenic factors (43). Moreover, atorvastatin was found to inhibit the activity of breast cancer cells via inducing autophagy (44). In addition, lovastatin could mediate MCF-7 cancer cell death by interaction with p53-survivin signaling cascade (45). Taken together, the mechanisms underlying the potential benefits of statins in breast cancer are likely to be multifactorial, and further studies are warranted to determine the key molecular pathway involved.

Our study has limitations, which should be considered when interpreting the results. Firstly, although we combined HR data after multivariate adjustment, residual factors that potentially confound the association between statin use and prognosis in breast cancer may remain existing. Secondly, definition and exposure time of statin use varied among the included studies. Although our stratified analyses did not show that timing, category, or exposure time of statin use may significantly affect the outcome, these results should be validated in randomized clinical trials. In addition, our results of subgroup analyses were based on data of study level rather than individual patient level. The findings of subgroup analyses should be validated in large-scale prospective studies. Finally, a causative relationship between statin use and improved prognosis in women with breast cancer should not be retrieved from our results. Randomized clinical trials are needed to confirm whether additional treatment with statin could improve the clinical outcomes in women with breast cancer.

In conclusion, our meta-analysis showed that statin use was associated with significant reduced recurrence and disease-specific mortality in women with breast cancer. These findings support the implementation of a randomized clinical trial to evaluate the potential benefits of statins on clinical outcomes in women with breast cancer.

## Data Availability Statement

All datasets generated for this study are included in the article/supplementary files.

## Author Contributions

HL and PH designed the study and drafted the manuscript. HL and DS performed database search, study inclusion, quality evaluation, and data extraction. MF, YC, FX, ZW, and YW performed statistical analyses and interpreted the data. All authors critically reviewed the manuscript and approved its submission.

## Conflict of Interest

The authors declare that the research was conducted in the absence of any commercial or financial relationships that could be construed as a potential conflict of interest.
